# Editorial: Value of a multidisciplinary approach for modern diagnosis of infectious diseases

**DOI:** 10.3389/fcimb.2024.1514207

**Published:** 2024-11-15

**Authors:** Andrea Marino, Stefano Stracquadanio, Stefano Marletta

**Affiliations:** ^1^ Department of Clinical and Experimental Medicine, Unit of Infectious Diseases, ARNAS Garibaldi Hospital, University of Catania, Catania, Italy; ^2^ Department of Biomedical and Biotechnological Sciences, Section of Microbiology, University of Catania, Catania, Italy; ^3^ Division of Pathology, Humanitas Cancer Center, Catania, Italy; ^4^ Department of Diagnostics and Public Health, Section of Pathology, University of Verona, Verona, Italy

**Keywords:** precision medicine, artificial intelligence, infectious disease, microbiology, pathology

## Editorial

Infectious diseases continue to pose significant challenges to global public health, exacerbating the burden on healthcare systems worldwide (Naghavi et al., 2024; La Via et al., 2024). The rise of antimicrobial resistance (AMR), the emergence of new pathogens, and the re-emergence of familiar ones have created an urgent need for innovative solutions in diagnosis and treatment. Infectious agents such as bacteria, viruses, fungi, and parasites cause a broad spectrum of diseases that account for a considerable percentage of global morbidity and mortality (Charalampous et al., 2023). Early and accurate diagnosis is essential for controlling outbreaks, improving patient outcomes, and curbing the misuse of antibiotics, which fuels resistance (Hahn et al., 2020; Saydam et al., 2021).

Traditional diagnostic methods, though valuable, often struggle to keep pace with the rapid evolution of pathogens and the complexity of infectious diseases (Vidyarthi et al., 2022). Techniques such as culture-based tests, even if reliable, are slow and can lack the sensitivity needed for early detection (Miller et al., 2024). Empirical treatments, frequently employed due to diagnostic delays, may be ineffective and contribute to resistance. In this context, precision diagnostics and novel therapeutic strategies have emerged as pivotal tools in infectious disease management, offering faster, more accurate, and more individualized approaches to tackling infections (Devrim et al., 2022).

Recent advances in molecular biology, bioinformatics, and biotechnology have given rise to precision diagnostic tools capable of addressing the shortcomings of conventional methods (Liu et al., 2023). Technologies like next-generation sequencing (NGS), aptamer-based diagnostics, and advanced immunoassays are revolutionizing the way we diagnose infections. These tools can detect pathogens with greater sensitivity and specificity, even in cases where traditional methods fall short. Furthermore, the advent of real-time, point-of-care diagnostic systems (Stracquadanio et al., 2021) has made it possible to deliver rapid, actionable results, transforming the way clinicians approach treatment.

At the intersection of diagnostics and therapeutics, precision tools are also playing a crucial role in developing targeted treatments. Aptamers, for example, are nucleic acid-based molecules that can be engineered to bind specific pathogens or toxins, offering a highly selective and effective means of both diagnosing and neutralizing infections. Additionally, technologies such as metagenomics and sonication protocols are expanding our understanding of microbial communities and biofilms, leading to more effective interventions against chronic and resistant infections.

This Research Topic brings together ten articles that explore cutting-edge developments in the use of precision tools for diagnosing and treating infectious diseases. From advancements in rapid diagnostic assays to innovative approaches in therapeutic intervention, the contributions in this Research Topic illustrate the potential of these tools to reshape the landscape of infectious disease management. Whether addressing the diagnostic challenges of bloodstream infections, optimizing the identification of pathogens in surgical site infections, or exploring novel diagnostic markers for rare diseases, the research presented here is a testament to the progress being made in the field.

By integrating precision diagnostics and targeted therapies, we are entering a new era in infectious disease management—one where personalized care can lead to better outcomes, reduced transmission, and more efficient use of resources. As we move forward, continued research and innovation in this domain will be critical in our ongoing efforts to control and eventually eliminate the global threat of infectious diseases.

## Advancements in diagnostic technologies

The shift toward rapid, accurate diagnostic techniques is a focal point of this Research Topic. Aptamer-based technologies, as discussed by Sujith et al., offer a promising approach by providing high specificity and sensitivity, surpassing traditional antibody-based methods. Their structural robustness makes them highly suitable for long-term storage and application in molecular diagnostics. Similarly, the study by Xiao et al. presents a novel real-time fluorescent multiple cross displacement amplification technique for *Mycoplasma pneumoniae* detection, providing a rapid and sensitive alternative for point-of-care settings, which could prove invaluable in resource-limited regions.

## Next-generation sequencing and metagenomics

Metagenomic next-generation sequencing (mNGS) is revolutionizing the way bloodstream infections (BSIs) are diagnosed. Yu et al. highlight the advantages of mNGS in detecting pathogens in plasma samples, showing superior performance compared to traditional blood cultures, particularly for gram-negative bacteria. This method represents a critical step forward in reducing diagnostic delays and improving outcomes in sepsis management.

## Therapeutic implications and resistance challenges

The emergence of drug-resistant pathogens, such as carbapenem-resistant *Klebsiella oxytoca* in emergency neurosurgical settings, as explored by Jiang et al., underscores the importance of timely and precise interventions. The study traces the transmission of resistant bacteria, identifying the contamination source and advocating for standardized infection control procedures (El-Sokkary et al., 2022; Marino et al., 2023).

Similarly, the research by Zhang et al. on *Nocardia otitidiscaviarum* infection demonstrates the life-saving potential of early diagnosis and tailored treatments in managing rare but deadly infections. This case further emphasizes the role of advanced laboratory techniques, such as metagenomic sequencing and mass spectrometry, in diagnosing complex infectious diseases.

## Emerging diagnostic tools

Innovative diagnostic assays, such as the chemiluminescence-based urinary lipoarabinomannan (LAM) antigen assay for tuberculosis detection, are advancing the field. Huang et al. report the assay’s high specificity for extrapulmonary tuberculosis (EPTB), offering a non-invasive and efficient diagnostic tool that could reduce mortality, especially in settings where conventional methods fall short.

Moreover, the development of a sandwich immunoassay targeting *Francisella tularensis* outer membrane protein A for tularemia diagnosis, as described by Jang et al., represents a leap forward in addressing bioterrorism-related pathogens. This assay’s high sensitivity and specificity could have far-reaching applications in public health and biodefense.

## Future directions and broader implications

The articles in this Research Topic collectively highlight the necessity of adopting precision tools in diagnosing and treating infectious diseases. From real-time diagnostics to the identification of resistance mechanisms, the work presented here advances our understanding of pathogen detection and management. Looking ahead, the integration of these novel technologies with further technological developments such as artificial intelligence-based tools (Marletta et al., 2023) into clinical practice will be crucial in addressing the growing threat of infectious diseases and antimicrobial resistance.

We extend our sincere gratitude to all the contributors for their valuable insights and groundbreaking research, and we hope this Research Topic will inspire further advancements in the fight against infectious diseases.

## References

[B1] Charalampous P. Haagsma J. A. Jakobsen L. S. Gorasso V. Noguer I. Padron-Monedero A.. (2023). Burden of infectious disease studies in Europe and the United Kingdom: a review of methodological design choices. Epidemiol. Infect. 151, e19. doi: 10.1017/S0950268823000031 36621004 PMC9990389

[B2] Devrim I. Erdem H. El-Kholy A. Almohaizeie A. Logar M. Rahimi B. A.. (2022). Analyzing central-line associated bloodstream infection prevention bundles in 22 countries: The results of ID-IRI survey. Am. J. Infect. Control 50, 1327–1332. doi: 10.1016/j.ajic.2022.02.031 35263612

[B3] El-Sokkary R. Erdem H. Kullar R. Pekok A. U. Amer F. Grgić S.. (2022). Self-reported antibiotic stewardship and infection control measures from 57 intensive care units: An international ID-IRI survey. J. Infect. Public Health 15, 950-954. doi: 10.1016/j.jiph.2022.07.009 35917656

[B4] Hahn A. Podbielski A. Meyer T. Zautner A. E. Loderstädt U. Schwarz N. G.. (2020). On detection thresholds-a review on diagnostic approaches in the infectious disease laboratory and the interpretation of their results. Acta Trop. 205, 105377. doi: 10.1016/j.actatropica.2020.105377 32007448

[B5] La Via L. Sangiorgio G. Stefani S. Marino A. Nunnari G. Cocuzza S.. (2024). The global burden of sepsis and septic shock. Epidemiol. (Basel Switzerland) 5, 456–478. doi: 10.3390/epidemiologia5030032 PMC11348270 39189251

[B6] Liu Q. Jin X. Cheng J. Zhou H. Zhang Y. Dai Y. (2023). Advances in the application of molecular diagnostic techniques for the detection of infectious disease pathogens (Review). Mol. Med. Rep. 27, 104. doi: 10.3892/mmr.2023.12991 37026505 PMC10086565

[B7] Marino A. Stracquadanio S. Campanella E. Munafò A. Gussio M. Ceccarelli M.. (2023). Intravenous fosfomycin: A potential good partner for cefiderocol. Clinical experience and considerations. Antibiotics 12 (1), 49. doi: 10.3390/antibiotics12010049 PMC9854867 36671250

[B8] Marletta S. L’Imperio V. Eccher A. Antonini P. Santonicco N. Girolami I.. (2023). Artificial intelligence-based tools applied to pathological diagnosis of microbiological diseases. Pathol. Res. Pract. 243, 154362. doi: 10.1016/j.prp.2023.154362 36758417

[B9] Miller J. M. Binnicker M. J. Campbell S. Carroll K. C. Chapin K. C. Gonzalez M. D.. (2024). Guide to utilization of the microbiology laboratory for diagnosis of infectious diseases: 2024 update by the infectious diseases society of america (IDSA) and the american society for microbiology (ASM). Clin. Infect. Dis. 5, ciae104. doi: 10.1093/cid/ciae104 38442248

[B10] NaghaviM.VollsetS. E.IkutaK. S.SwetschinskiL. R.GrayA. P.WoolE. E.. (2024). Global burden of bacterial antimicrobial resistance 1990-2021: a systematic analysis with forecasts to 2050. Lancet (London England) 404, 1199–1226.39299261 10.1016/S0140-6736(24)01867-1

[B11] Saydam F. N. Erdem H. Ankarali H. El-Arab Ramadan M. E. El-Sayed N. M. Civljak R.. (2021). Vector-borne and zoonotic infections and their relationships with regional and socioeconomic statuses: An ID-IRI survey in 24 countries of Europe, Africa and Asia. Travel Med. Infect. Dis. 44, 102174. doi: 10.1016/j.tmaid.2021.102174 34699956

[B12] Stracquadanio S. Di Gaudio F. Giunta E. Falliti G. Caruso D. Pinzone C.. (2021). Reverse transcriptase loop-mediated isothermal amplification (RT-LAMP) as a user-friendly system to detect SARS-CoV-2 infection: a multicentric study. New Microbiol. 44, 181–183. Available at. 34783350

[B13] Vidyarthi A. J. Das A. Gupta A. (2022). Revisiting conventional microbiology techniques in the era of molecular testing. Curr. Med. Res. Pract. 12, 274–279. doi: 10.4103/cmrp.cmrp_60_22

